# Evaluation of the experiences and needs of users of a drug information resources website

**DOI:** 10.5195/jmla.2020.446

**Published:** 2020-04-01

**Authors:** Jennifer E. Isenor, Melissa Helwig, Michael B. Weale, Susan K. Bowles

**Affiliations:** Associate Professor, College of Pharmacy, Dalhousie University, Halifax, NS, Canada, jennifer.isenor@dal.ca, https://orcid.org/0000-0003-1648-7362; Librarian, W.K. Kellogg Library, Dalhousie University, Halifax, NS, Canada, helwig@dal.ca, https://orcid.org/0000-0001-9915-3928; Shoppers Drug Mart, Canada, m.weale@dal.ca; Associate Professor, Nova Scotia Health Authority and Dalhousie University, Halifax, NS, Canada, susan.bowles@nshealth.ca, https://orcid.org/0000-0003-0821-3222

## Abstract

**Objective:**

This article describes the evaluation of the experiences and needs of users of the Drug Information Resources (DIR) website. The DIR website attracts traffic and use from around the world, with the highest number of users in Canada and the United States.

**Methods:**

An online questionnaire was developed through use of a literature review and Google Analytics data. Face validity testing and test-retest reliability were completed prior to releasing the questionnaire.

**Results:**

Although the Google Analytics data showed that the site is used internationally, most respondents were Canadian students. They used the site for academic and clinical purposes and reported it was easy to use, was well organized, and included required resources, and they would recommend it to others.

**Conclusion:**

The DIR website was found to be a valuable resource for educational and clinical use. Future studies will aim to obtain input from international users.

## INTRODUCTION

The ability to seek, find, and utilize information at the point of need is a crucial element of evidence-based health care practice. Health care professionals’ clinical decision-making and patient care are impacted by the availability of many electronic resources of varying quality [1–4]. The Internet has made information more readily available to health care practitioners and their patients, but with this ease of access, users experience difficulties in identifying and filtering “the most useful, accurate, and credible sources while searching online for health information” [5]. For example, a 2015 survey found that 94% of community pharmacists in Tennessee used only electronic resources, including apps such as Epocrates and Lexicomp [6]. The results of that survey indicated that pharmacists were able to identify the gap in their resource use (most often related to primary resource availability), and the authors suggested that training could help pharmacists engage with drug information resources [6]. In other examples, surveys of medical residents and nurses showed that searches for information most frequently involved Google and Wikipedia, in addition to UpToDate and PubMed [7, 8].

Beyond considering the particular resources used, these studies show that health care professionals lack training in searching for information and knowing where to begin a search [7–9]. Furthermore, although clinicians have a need for quality information, they also lack time to search, suggesting that being able to quickly access and grasp critical information can take precedence over the reliability of the information [8–14]. Thus, the increasing number of online resources and desire of health care professionals to quickly find information indicates a need for high-quality, reputable resources, such as the Drug Information Resources (DIR) website [2, 15–17].

The DIR website is a portal to credible and current health information on Dalhousie University’s College of Pharmacy website that has existed since 1996. The website was initially created as a guide for learners at the College of Pharmacy to help them seek and use quality resources. The website has grown over time and now receives more than 5,000 visits per month from users in over 150 countries. The website has more than 60 categories, including but not limited to drug monographs, compounding, policy, patient counselling, and health and disease state pages. Each page includes links to critically appraised resources that are held by or licensed through Dalhousie Libraries, freely available online, or available for purchase (e.g., databases not licensed through Dalhousie but available for purchase by individuals, such as UpToDate). The website is updated to ensure that the listed resources appeal to a general pharmacy and health care practice audience and meet the educational needs of pharmacy students.

The DIR website is not only recognized by the local community, but is also recommended by the PharmGuide, which is maintained by members of the Pharmacy and Drug Information Section of the Medical Library Association, members of the Special Libraries Association, and individual members of the American Association of Colleges of Pharmacy [18] and the American Association of College of Pharmacy Basic Resources for Pharmaceutical Education [19]. A pharmacy LibGuide has since been developed [20], but the LibGuide focuses on the curriculum-related needs of Dalhousie pharmacy students, whereas DIR is for all users, including pharmacy and non-pharmacy students and clinicians. Although the websites have some overlap, they generally complement each other.

Although the DIR website has been in use since 1996, no formal studies have sought feedback from users, resulting in a gap in knowledge about site traffic and the actual experiences and needs of users. Therefore, the goal of this study was to evaluate the experiences and needs of users of the DIR website.

## METHODS

A mixed methods approach using an online questionnaire and focus groups with local DIR users was employed. While the questionnaire was intended for both local and international users, the focus groups were intended to ensure the site met the needs of local users. Both the questionnaire and focus groups were deemed quality assurance and exempt from review by the Dalhousie University Health Sciences Research Ethics Board.

### Questionnaire

The online questionnaire was developed using Google Analytics data for the DIR website and a review of the literature on usability and information portal practices. Face validity testing was completed with a convenience sample of 12 people from DIR user groups, including health care professionals, health professions students, university faculty, and librarians. Test-retest reliability of the updated questionnaire was evaluated with a convenience sample of 9 people from these DIR user groups, using the intraclass correlation coefficient, and was >0.9 for all questions, indicating a high degree of reliability.

The self-administered online questionnaire (supplemental appendix) was administered using Opinio survey software [21]. A link to the questionnaire was added to the DIR website from October 2014 to May 2015. Social media, emails to stakeholder groups (e.g., pharmacy associations), and in-person presentations to students were used to increase interest in completing the questionnaire. Questionnaire responses were analyzed using XLSTAT for Windows 10.0 (Microsoft Corporation). Summary statistics were used to describe demographic characteristics of DIR users and their responses to questions regarding user experience.

### Focus groups

Responses to the online questionnaire were used to inform questions for the focus groups. Three semi-structured focus group sessions were held in the fall of 2015 with clinicians, faculty, librarians, and students. Participants were recruited via posters around Dalhousie, student and faculty email discussion lists, and convenience sampling of local librarians and clinicians. Focus groups were audio-recorded and reviewed for codes and themes.

## RESULTS

### Questionnaire

A total of 114 questionnaires were completed. Most respondents were students (n=102, 89%), with 55 (48%) reporting to be pharmacy students, 1 (1%) a science student, and 46 (40%) not indicating their area of study. Other respondents included health care professionals (n=11, 10%) and a faculty member (n=1, 1%). Respondents were mainly from Canada (n=107, 94%), with 1 (1%) from the United States and 7 (6%) who did not report where they lived. From October 2014 to May 2015, DIR web traffic, based on Google Analytics, indicated that 89% of users accessed the DIR website from Canada and 2% from the United States, similar to the respondent group. DIR web traffic indicated that 80% of visitors to DIR were returning users, while 20% were new users to the site.

Most respondents (76%) reported that they found the information they sought on the DIR website, with 3% reporting they did not and 21% not responding. Overall, most respondents were satisfied with the DIR website, finding it easy to use, visually appealing, and well organized (Table 1). Although most people did not provide a response or indicated that they did not use DIR for questions related to clinical information or decision making by choosing “not applicable,” those who indicated the frequency of their DIR use for clinical decision making generally reported weekly to monthly use depending on the category listed (Table 2).

**Table 1:** Survey respondents’ satisfaction with the Drug Information Resources (DIR) website (n=114)

	Strongly agree		Agree		Uncertain		Disagree		Strongly disagree		Not applicable		Not reported	

	n	(%)	n	(%)	n	(%)	n	(%)	n	(%)	n	(%)	n	(%)
The website is easy to use upon my first visit	36	(32%)	58	(51%)	3	(3%)	2	(2%)	0	(−)	2	(2%)	13	(11%)
The website is visually appealing	30	(26%)	55	(48%)	7	(6%)	5	(4%)	1	(1%)	2	(2%)	14	(12%)
Information is clearly organized on the website	33	(29%)	54	(47%)	4	(4%)	7	(6%)	1	(1%)	2	(2%)	13	(11%)
Topic pages (e.g., compounding) are well designed	24	(21%)	60	(53%)	12	(10%)	1	(1%)	1	(1%)	2	(2%)	13	(11%)
Terminology used in the website is clear	42	(37%)	54	(47%)	1	(1%)	1	(1%)	0	(−)	2	(2%)	14	(12%)
Content on the website met my expectations	40	(35%)	55	(48%)	1	(1%)	1	(1%)	1	(1%)	2	(2%)	14	(12%)
I found the website cumbersome to use	8	(7%)	8	(7%)	10	(9%)	52	(46%)	18	(16%)	4	(3%)	13	(11%)
I am able to find the information I need easily on this site	29	(25%)	62	(54%)	5	(4%)	3	(3%)	0	(−)	2	(2%)	13	(11%)
Clicking on the links takes me to what I expect	40	(35%)	53	(46%)	2	(2%)	3	(3%)	1	(1%)	2	(2%)	13	(11%)
I would use this website in the future	73	(64%)	26	(23%)	1	(1%)	0	(−)	0	(−)	1	(1%)	13	(11%)
Overall, the website is easy to use	47	(41%)	46	(40%)	4	(4%)	2	(2%)	0	(−)	2	(2%)	13	(11%)

**Table 2:** Survey respondents’ indications of how frequently they carry out the following actions as a result of obtaining clinical information from the DIR website (n=114)

Action	Daily		Weekly		Monthly		Every few months		Yearly		Never		Not applicable		No response	

	n	(%)	n	(%)	n	(%)	n	(%)	n	(%)	n	(%)	n	(%)	n	(%)
Change or recommend a change in a patient’s medication(s)	7	(6%)	20	(18%)	9	(8%)	2	(2%)	0	(−)	3	(3%)	48	(42%)	25	(22%)
Print out information for a patient or recommend a website to a patient	2	(2%)	11	(10%)	17	(15%)	10	(9%)	1	(1%)	5	(4%)	43	(38%)	25	(21%)
Recommend a behavior modification or change of habits (e.g., lifestyle) to a patient	4	(3%)	17	(15%)	11	(10%)	7	(6%)	0	(−)	5	(4%)	46	(40%)	24	(21%)
Modify or recommend a modification to a patient’s treatment or therapy	6	(5%)	17	(15%)	11	(10%)	6	(5%)	0	(−)	2	(2%)	46	(40%)	26	(23%)
Request or recommend further tests or a referral	3	(3%)	5	(4%)	12	(11%)	6	(5%)	5	(4%)	10	(9%)	49	(43%)	24	(21%)
Request more information about a product or medication	11	(10%)	13	(11%)	12	(11%)	8	(7%)	4	(4%)	10	(9%)	33	(29%)	23	(20%)
Conduct further research using other resources	13	(11%)	24	(21%)	14	(12%)	8	(7%)	2	(2%)	6	(5%)	23	(20%)	24	(21%)

The questionnaire included open-ended questions about what the strengths of the DIR website are, what new content or features they would like added to it, what they would suggest to improve it, and if they would recommend it to others. Respondents indicated that the strengths of DIR were its ease of use, comprehensive coverage of resources, and organization or layout. Respondents suggested specific resources, such as UpToDate and Pharmacist’s Letter (two resources that are not available through the local university), and content categories, including drug pricing resources, more drug interaction checkers, and links to more mobile apps. Suggested areas of improvement included updates to the index and improvement of the search function. Two main themes emerged around why respondents would recommend the site to others: (1) a large amount of information that is available in one place and (2) ease of use.

### Focus groups

A total of twenty-five participants attended the focus groups, most of which were students. All focus group participants indicated that they found the DIR website to be a valuable resource. Students indicated they used it daily for school and clinical practice (e.g., clinical rotations or part-time jobs). Students noted they bookmarked the site and preferred using the “Quick Links” on the “Dal Access” home page. Although they were unaware of the “Research” resources section, when it was shown to participants during the focus groups, they indicated that it would be useful for coursework. Health On the Net (HON) certification [22] was not considered to be important as they already trusted the website, although they recognized it might be useful for those who were not affiliated with Dalhousie.

## DISCUSSION

The study results indicated that local student users valued the DIR website, used it for academic and clinical work, and had minimal suggestions for areas of improvement, although they indicated they wanted to see it continue to exist and be updated. Although the research did not meet the initial objective to evaluate the site for all users, formal feedback from DIR users had not been previously sought; therefore, this study provided insight on how local users (learners and practitioners) perceived the site, the types of information they used on the site, and the types of questions they might use information from the site to answer.

Many respondents did not respond or selected “not applicable” to questions related to the frequency of use of the DIR website for clinical decision making and patient care, so specific conclusions could not be made. This result could be due to the high volume of student respondents using the site for school-related work. The Institute for Safe Medication Practices states that access to accurate and usable drug information as well as a variety of resources is important for reducing preventable adverse drug events [23], and it is encouraging to see that students are using DIR, which provides access to a variety of credible resources and may, thus, improve clinical decision making in practice. Student learners in a study on the NICE Evidence search indicated that they found that particular resource increasingly relevant as it became more familiar to them [24], and this could also be the case with the clinical decision making and patient care resources on the DIR with continued use.

The cost of maintaining a current portal can be a challenge, given shrinking library and research budgets. The website previously held HON certification, but due to the introduction of fees associated with HON certification, it was no longer possible to maintain its certification. Therefore, it was reassuring that focus group participants indicated that they trusted the website regardless and did not see a need for HON certification. One area in which budgets were a concern was the lack of institutional access to all desired resources, such as UpToDate and Pharmacist’s Letter.

As it is not feasible to have all resources, most sections of the DIR website offer links to a variety of freely available, institutionally available, and private-pay resource options. Links to resources that are not funded are listed on the website, and individuals are redirected to opportunities to purchase materials or subscriptions, if they desire. Although the resources might not be available at Dalhousie, users outside of Dalhousie may have access to them, so in this case, DIR acts as a guide to direct users to other resources they can use. The DIR website can also help guide Dalhousie graduates to resources that they may not have used during their time at Dalhousie but to which they have access in their workplace.

Strengths of this study included using a review of the literature and Google Analytics data to develop the questionnaire, as well as completion of face validity testing and test-retest reliability. The validated questionnaire (supplemental appendix) that was used in this study can be adapted by others to assess their information resource websites. While the number of online resources and apps continues to increase, there are concerns about information overload and the time required to interpret and appraise the resources [2, 4–6, 16, 17]. The questionnaire results provide evidence that the DIR website is a “reliable and useful web-based resource,” which Grossman and Zerilli suggest can assist health care professionals “expedite the information gathering and decision-making process” [17]. Therefore, health care professionals may want to bookmark the site to consult when they respond to information requests, as it provides access to freely available resources and links to licensed resources. The results of the survey also provide further validation of why the site continues to be recommended internationally [18, 19].

One limitation of this study was the low number of responses. Despite this limitation, the demographics of respondents were proportional to DIR web traffic with respect to geographic location according to Google Analytics. In addition, Google Analytics data indicated a high percentage of return visitors, which is further supported by the majority of respondents’ indication that they would use the site again. Although it is important to note that Google Analytics do not take into account the same person using different Internet protocol (IP) addresses (e.g., desktop, laptop, tablet, smartphone, or home versus work or school), it does provide an estimate of user activity. Another limitation was that the research team was unable to evaluate the site for all users due to the small response rate and the majority of respondents being from Canada. Therefore, a goal for future research will be to engage nonlocal users of the DIR website for input on what would improve the site for them. Another limitation was the number of respondents who did not provide an answer or indicated “not applicable” when asked about use of DIR for clinical information and decision making, which might be due to the large proportion of student respondents who might not have been working clinically, because the survey was available during the school year.

The DIR website was found to be a valuable resource for educational and, in some cases, clinical use locally. The development and validation of the questionnaire for evaluating portals like DIR can be customized and used by others to gather input on their information resource websites. The provided feedback was used to improve the website and added evidence of DIR’s credibility. In addition, the results will be shared with other educators and professionals regarding learners’ resource use and further applied to the curriculum related to information resources. Going forward, the DIR website will continue to focus on the promotion and use of evidence-based medicine resources. Future research will aim to better capture input from DIR users outside of the local region.

## SUPPLEMENTAL FILE

Appendix Questionnaire

## 

**Jennifer E. Isenor**, jennifer.isenor@dal.ca, https://orcid.org/0000-0003-1648-7362, Associate Professor, College of Pharmacy, Dalhousie University, Halifax, NS, Canada

**Melissa Helwig**, helwig@dal.ca, https://orcid.org/0000-0001-9915-3928, Librarian, W.K. Kellogg Library, Dalhousie University, Halifax, NS, Canada

**Michael B. Weale**, m.weale@dal.ca, Shoppers Drug Mart, Canada

**Susan K. Bowles**, susan.bowles@nshealth.ca, https://orcid.org/0000-0003-0821-3222, Associate Professor, Nova Scotia Health Authority and Dalhousie University, Halifax, NS, Canada
