# Supplementary material for: Evaluation of the experiences and needs of users of a drug information resources website
Source: J Med Libr Assoc. 2020 Apr 1;108(2):270–7. doi: 10.5195/jmla.2020.446 (PMC7069831; doi:10.5195/jmla.2020.446)
Supplement: Appendix [file jmla-108-270-s001.pdf]

JMLA Journal of the Medical Library Association

SC

Supplemental content to
J Med Libr Assoc. Apr;108(2):dx.doi.org/10.5195/jmla.2020.446
www.jmla.mlanet.org
© Isenor, Helwig, Weale, Bowles 2020

Evaluation of the experiences and needs of users of a drug information resources website

Jennifer E. Isenor; Melissa Helwig; Michael B. Weale; Susan K. Bowles

APPENDIX

Questionnaire

Question 1: Consent

By clicking on the "Agree" button below, I consent to participate in this survey. I am aware that I am free to withdraw from the survey at any time up until I submit my responses to the survey.

- Agree
- Do not agree (When chosen, survey does not proceed, and a closing screen thanks them for their interest in the Drug Information Resources [DIR] website)

Question 2: I am a/an: (select all that apply)

- Student
- Health care professional
- Faculty member
- Librarian
- Other, please specify: ___________

Question 3 (Branch when "Student" was selected in Q2): Please indicate what type of student you are:

- Undergraduate, please specify your area of study: ______________________________
- Health care professional program, please specify your area of study: ______________
- Graduate/postgraduate studies, please specify your area of study: _______________

Question 4 (Branch when "Health care professional" was selected in Q2): Please indicate your field of practice.

- Pharmacy
- Medicine
- Nursing
- Other, please specify your field of practice: ______________

Question 5: Which device(s) do you use to access the DIR? (select all that apply)

- Desktop computer
- Laptop or netbook
- Mobile phone or smartphone
- Tablet
- Other, please specify: ______________

Question 6: Are you located in Canada?

- Yes (break out to provinces)
  - Question 7: Which province are you located in? ______________
- No; please specify your country: ______________

Journal of the Medical Library Association

Supplemental content to
J Med Libr Assoc. Apr;108(2):dx.doi.org/10.5195/jmla.2020.446
www.jmla.mlanet.org
© Isenor, Helwig, Weale, Bowles 2020

Question 8: On average, how often do you use the following for seeking drug information:

	Daily	Weekly	Monthly	Every few months	Yearly	Never
Internet						
Wikipedia						
Ask a colleague						
University or library resources (excluding DIR)						
DIR website						

Question 9: When do you use DIR? (select all that apply)
- o When I require an urgent answer (i.e., within 20 minutes)
- o When I require an immediate answer (i.e., within 1 day)
- o When I require a non-immediate answer (i.e., greater than 1 day)
- o When I am searching an area of interest, without a specific clinical question or time frame
- o Other, please specify: ______________

Question 10: How much time can you/are you willing to spend on trying to find the answer to a clinical question on DIR before trying another resource?
- o Less than 10 minutes
- o 10 to 20 minutes
- o 20 to 30 minutes
- o More than 30 minutes

Question 11: How did you learn about the DIR website? (select all that apply)
- o From an instructor/professor
- o Suggested by a colleague, classmate, or friend
- o Linked from another website
    - – Question 12: Which website linked or directed you to DIR? ______________
- o Web search (e.g., Google search)
- o Other, please specify: ______________

SC
Supplemental content to
J Med Libr Assoc. Apr;108(2):dx.doi.org/10.5195/jmla.2020.446
www.jmla.mlanet.org
© Isenor, Helwig, Weale, Bowles 2020

Question 13: Please indicate your agreement with the following statements regarding DIR.

	Strongly agree	Agree	Uncertain	Disagree	Strongly disagree	NA
The website is easy to use upon my first visit						
The website is visually appealing						
Information is clearly organized on the website						
Topic pages (e.g., Compounding) are well designed						
Terminology used in the website is clear						
Content on the website met my expectations						
I found the website cumbersome to use						
I am able to find the information I need easily on this site						
Clicking on the links takes me to what I expect						
I would use this website in the future						
Overall, the website is easy to use						

Question 14: How do you plan to use the information you found on the DIR website? (select all that apply)

- General information
- Patient/health care recommendations
- Review resources to purchase
- Review resources for inclusion in course syllabi
- To complete school assignments/tasks
- Other, please specify: ______________

JMLA Journal of the Medical Library Association

SC

Supplemental content to
J Med Libr Assoc. Apr;108(2):dx.doi.org/10.5195/jmla.2020.446
www.jmla.mlanet.org
© Isenor, Helwig, Weale, Bowles 2020

Question 15: How often do you carry out the following actions as a result of obtaining clinical information from DIR?

	Daily	Weekly	Monthly	Every few months	Yearly	Never	NA
Change or recommend a change in a patient's medication(s)							
Print out information for a patient or recommend a website to a patient							
Recommend a behaviour modification or change of habits (e.g., lifestyle) to a patient							
Modify or recommend a modification to a patient's treatment/therapy							
Request or recommend further tests or a referral							
Request more information about a product or medication							
Conduct further research using other resources							

Question 16: While utilizing DIR, did you find the information and/or resource that you needed?

- o Yes
- o No
  - – Question 17: If your need was not met by DIR, what other resource(s) did you use? ______________

Question 18: What are the greatest strengths of the DIR website?

Question 19: What new content or features would you like to see added to DIR in the future?

JMLA Journal of the Medical Library Association

SC
Supplemental content to
J Med Libr Assoc. Apr;108(2):dx.doi.org/10.5195/jmla.2020.446
www.jmla.mlanet.org
© Isenor, Helwig, Weale, Bowles 2020

Question 20: Do you have any other suggestions for ways to improve the DIR website?

Question 21: Would you recommend the DIR site to others?
- o Yes
- o No

Please explain why or why not?

Question 22: Thank you for taking the time to complete this questionnaire. Please feel free to make any additional comments:
